# Upper Extremity Compartment Syndrome in a Patient with Acute Gout Attack but without Trauma or Other Typical Causes

**DOI:** 10.1155/2018/3204714

**Published:** 2018-01-23

**Authors:** John G. Skedros, James S. Smith, Marshall K. Henrie, Ethan D. Finlinson, Joel D. Trachtenberg

**Affiliations:** ^1^Department of Orthopaedic Surgery and Utah Orthopaedic Specialists, The University of Utah, 5323 South Woodrow Street, Salt Lake City, UT 84107, USA; ^2^St. Marks Hospital, Salt Lake City, UT, USA

## Abstract

We report the case of a 30-year-old Polynesian male with a severe gout flare of multiple joints and simultaneous acute compartment syndrome (ACS) of his right forearm and hand without trauma or other typical causes. He had a long history of gout flares, but none were known to be associated with compartment syndrome. He also had concurrent infections in his right elbow joint and olecranon bursa. A few days prior to this episode of ACS, high pain and swelling occurred in his right upper extremity after a minimal workout with light weights. A similar episode occurred seven months prior and was attributed to a gout flare. Unlike past flares that resolved with colchicine and/or anti-inflammatory medications, his current upper extremity pain/swelling worsened and became severe. Hand and forearm fasciotomies were performed. Workup included general medicine, rheumatology and infectious disease consultations, myriad blood tests, and imaging studies including Doppler ultrasound and CT angiography. Additional clinical history suggested that he had previously unrecognized recurrent exertional compartment syndrome that led to the episode of ACS reported here. Chronic exertional compartment syndrome (CECS) presents a difficult diagnosis when presented with multiple symptoms concurrently. This case provides an example of one such diagnosis.

## 1. Introduction

We report a case where a severe gout attack and acute compartment syndrome (ACS) of the upper extremity occurred simultaneously in a 30-year-old male. The compartment syndrome was not associated with trauma or other typical causes. The patient also had ipsilateral forearm cellulitis and culture-proven infections of the elbow joint and olecranon bursa. The temporal association of a gout flare with ACS and the concurrent infections confused all clinicians that cared for the patient in the hospital setting and in clinics several months thereafter. Because of this, the patient's workup was complex, reflecting a broad differential diagnosis. He eventually provided sufficient descriptions of prior episodes of bilateral upper extremity swelling and pain that allowed them to be clearly differentiated from his gout attacks. These episodes were more consistent with CECS, with the worst case being the episode of ACS that we report here. CECS is a less common cause of compartment syndrome in the upper extremity, and to our knowledge, its coexistence with a severe gout attack has not been reported. It is important to note in this case report that it was mistakenly concluded that the patient had an initial one-time episode of ACS. Consequently, we describe the episode as ACS because this reflected the understanding that we had during the patient's September to November 2016 hospitalizations. Months later, it was understood that the episode of ACS was most likely the worst of a series of CECS episodes.

## 2. Case Report

The patient is a 30-year-old right-hand-dominant Polynesian (Tongan) male (height 182.9 cm, weight 141.9 kg, and BMI 42.6) with a long history of crystal-proven gout attacks. He had a chronic history of recurrent pain in his back, bilateral shoulders, elbows, hands/wrists, hips, knees, and ankles/feet. The patient had been prescribed febuxostat for chronic gout and hyperuricemia. Additional medical problems included a history of noninsulin dependent diabetes, but he was noncompliant with the medication prescribed for this. He is a two pack-a-day cigarette smoker and consumes alcohol only occasionally, but he denied consumption of alcohol for over one month. He also denied recent or past illicit drug use and had no prior history of local or systemic infections.

For four years, he was employed as a construction worker, which required heavy lifting, hammering nails, pulling cables, and other various repetitive activities. In August 2016, because of upper extremity swelling and joint pains caused by these work-related activities, he changed his manual work occupation to one that required less strenuous physical activity.

In mid-September 2016, he had been exercising with light weights for two consecutive days. He then noticed progressive swelling and pain in his right forearm, hand, and shoulder. The pain and moderate swelling persisted despite treatment with oral anti-inflammatory medications which had usually been sufficient to abate the majority of prior episodes of pain and swelling. The pain and swelling became tolerable when he reduced his activity level. Four days later, he was driving his car on a long trip, and after four hours had passed, he noted increased right shoulder pain with increased swelling of the right hand and forearm. He was treated in an emergency department (ED) with intravenous (i.v.) ketorolac tromethamine, oral prednisone and tramadol.

The following morning, he was seen in the ED of our hospital with worsening symptoms. He was admitted for a severe acute gout flare and emerging ACS of the right forearm and hand. He was afebrile but had what appeared to be right forearm/hand cellulitis. His blood pressure was 140/73, and heart rate was 89 beats/min. Results of lab tests performed during his hospitalization are summarized in Tables 1–3. Initial laboratory values included uric acid level of 7.7 (normal is <7.2), leukocytosis (13,800/*µ*l), and a normal blood glucose level of 84. However, his blood glucose values were elevated several times during the remaining seven-day hospitalization (99–162). His CKMB (creatine kinase-MB) was normal at 2.5 ng/ml (normal range 0.5–3.6), and CK was mildly elevated at 245 U/liter (normal range 35–224). He also tested negative for rheumatoid factor, thyroid abnormality, systemic lupus erythematous, and HIV-1 and HIV-2 antibodies. A blood toxicology screen was negative for narcotics, alcohol, and other elicit substances.

He was placed on colchicine, i.v. ketorolac tromethamine, and i.v. vancomycin (2,000 mg every 12 hours), and i.v. piperacillin/tazobactam (3.375 grams every six hours). A CT scan of his hand, forearm, and elbow revealed fluid accumulation in the right olecranon bursa and elbow joint. An MRI of his right shoulder did not reveal abnormalities. Needle aspirations of his right elbow joint, olecranon bursa, wrist, and shoulder were done. Aspiration of the right elbow joint revealed sodium urate crystals and had a white blood cell count of 91,650/mm^3^. Aspirations of the right wrist revealed calcium pyrophosphate (pseudogout), but no organisms grew from the wrist culture. The white blood cell count could not be determined from the wrist aspiration because the sample had clotted. CT angiogram and Doppler ultrasound tests of his right upper extremity were negative for vein or artery pathology.

The patient was taken urgently to surgery for compartment fasciotomies because of clinically obvious compartment syndrome, including tense volar compartments, reduced sensation in all fingers, reduced capillary refill, and severe pain with passive finger stretch [1, 2]. His blood pressure was 160/98 mmHg at the time of compartment pressure measurements, which were made immediately prior to the induction of general anesthesia. Using a Stryker® Intra-Compartmental Pressure Monitor Set (Kalamazoo, Michigan, USA), his compartment pressures were measured twice in each of three locations. The volar compartments were measured between the mid and distal forearm, and the values were 20 and 24 mmHg. The carpal tunnel region and the mid palm ulnar to the median nerve each measured 45–48 mmHg. The dorsal forearm measured 12 and 14 mmHg. The dorsal forearm was not more than mild/moderately swollen. In view of the clinical findings and volar pressures exceeding 30 mmHg [3, 4], surgical releases were done for only the volar compartments, including the carpal tunnel and all volar hand compartments [4].

**Figure 1:**
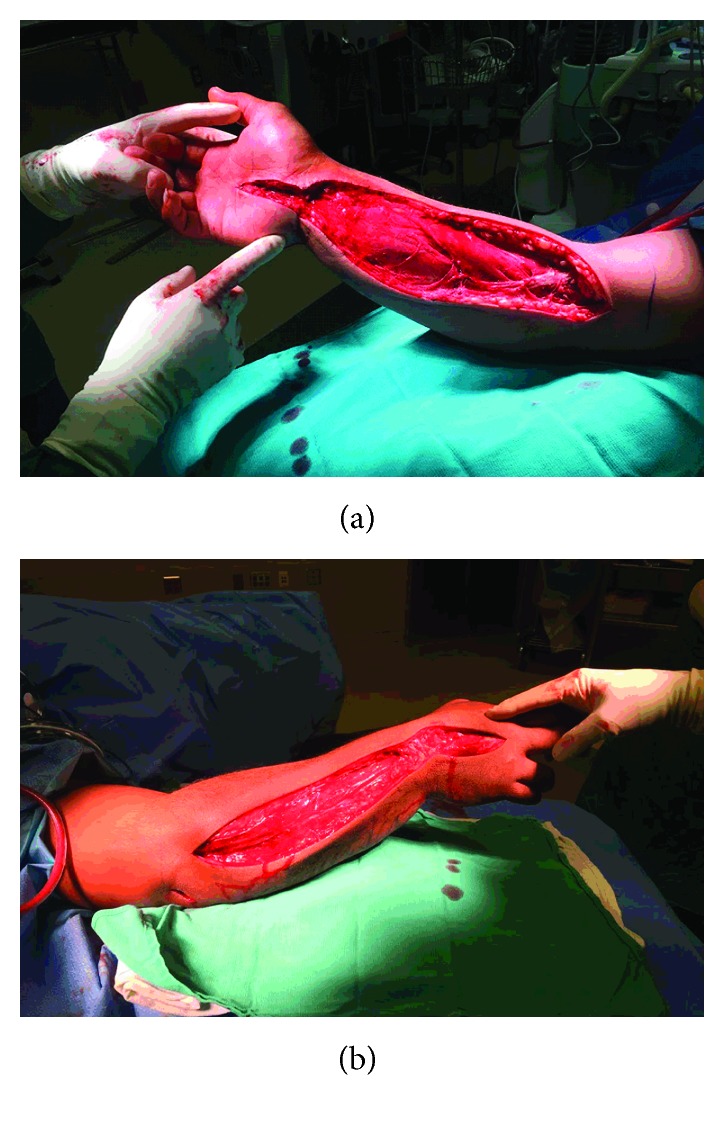
Intraoperative images of our patient's volar (a) and dorsal (b) fasciotomies of the right forearm and hand.

The volar forearm had bulging muscles that contracted with electrocautery stimulation, and there was no evidence of necrosis, pus, or odor (Figure 1). Gouty tophi were not grossly observed in the fascia or muscle, but small tophi (∼0.25–0.5 mm) were seen sporadically in the synovial sheaths of the flexor tendons, which can occur after many years in patients with poorly controlled gout [5]. The wounds were covered with sterile sponges attached to a conventional wound vacuum (V.A.C.Ulta™ Negative Pressure Wound Therapy System, KCI Medical, USA).

Because the swelling worsened over the 12 hours, especially in the dorsal hand and forearm, he was taken back to surgery, and new dorsal fasciotomies were made (Figure 1). The upper dorsal incision extended up to near the olecranon bursa, which allowed the superficial tissues to be elevated to access the olecranon bursa and elbow joint where infections were suspected in view of worsening white blood cell count and results of the elbow aspiration. The elbow joint was irrigated through a small arthrotomy and a bulb suction drain was placed.

Cultures from the tophitic/phlegmonous tissue obtained from the right olecranon bursa grew *Candida albicans*, which was treated with oral fluconazole. The fluid from the elbow aspiration grew *Staphylococcus haemolyticus*.

After a seven-day hospital stay, he was discharged to his home with four weeks of i.v. vancomycin and oral fluconazole and with outpatient wound vacuum sponge changes 2-3 times per week. Two weeks later, he was evaluated at a tertiary care hospital (University of Utah Medical Center) for a rheumatologic evaluation, which was not available at our hospital. In addition to having poorly controlled gout, he received the additional diagnosis of polyarthritis and oral prednisone and analgesics were recommended on an as-needed basis. At that time, all consulting physicians remained puzzled by the association of the patient's gout and joint pains with what was still considered a first-time episode of ACS.

**Figure 2:**
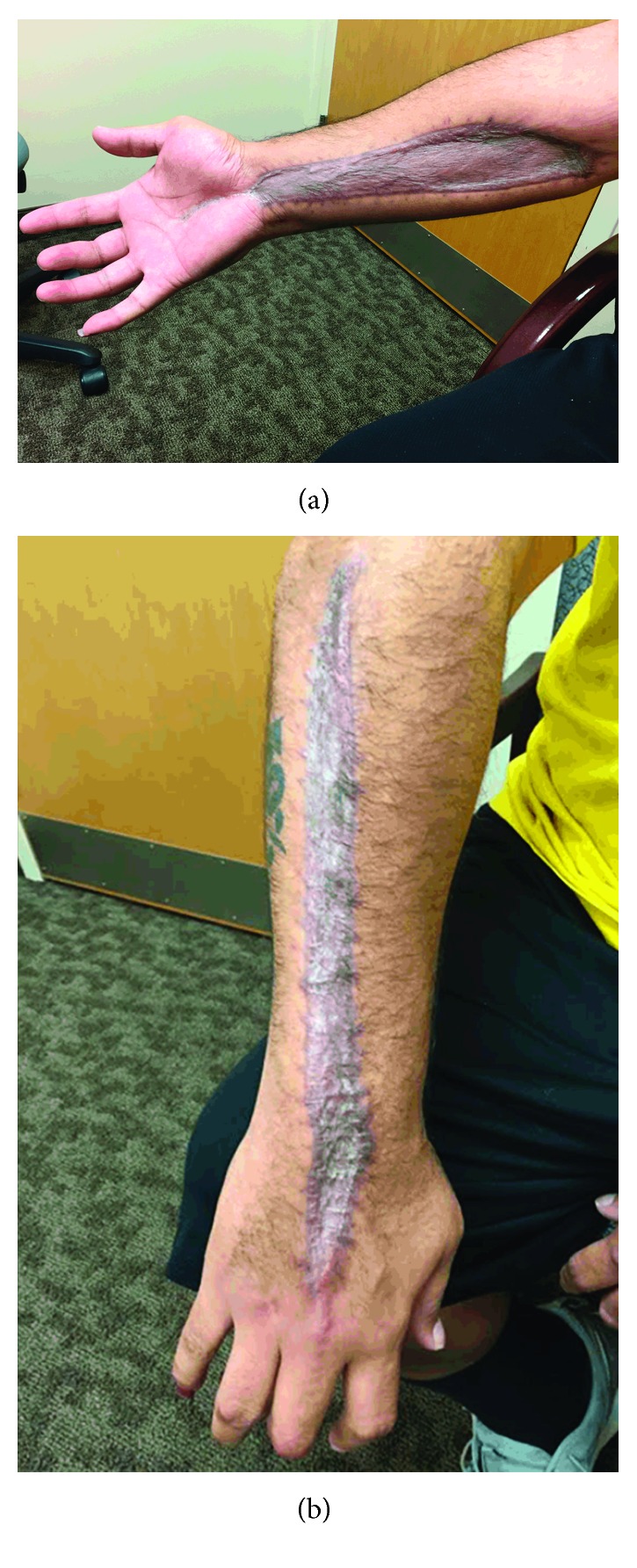
Three-month postoperative images showing healed skin grafts on the volar (a) and dorsal (b) aspects of our patient's right forearm and hand.

Split-thickness skin grafts were successfully performed (Figure 2). He was seen again in the ED of our hospital on two additional occasions for crystal-proven gout flares of his shoulders and knees without significant limb compartment swelling. His CK levels were not elevated, and the symptoms improved with colchicine and anti-inflammatory medications.

When he returned to our clinic three months later, we obtained more details of his prior “gout attacks,” including the duration, quality of pain and locations of swelling, and activities before their onset. It then became clear that what he believed, and had been told by all prior healthcare providers, were approximately 10 severe “gout attacks” over the past four years, which were more consistent with episodes of chronic exertional compartment syndrome (CECS) [1, 6, 7]. These episodes started several months after he began working in construction. Upon learning of the underlying diagnosis of CECS, the patient then stated that he was able to differentiate episodes of this condition from his gout flares because of what he considered to be distinct differences in pain and swelling intensity (gout-related pain and swelling was focused at the joint versus in CECS, the pain and swelling were of greater intensity and more diffuse in the limb).

At 12 months after the right upper extremity fasciotomies, the patient reported moderate weakness in grip strength and mild reduced motion of his finger joints. He did not return to manual labor and had no additional episodes of significant compartment swelling.

## 3. Discussion

This case is highly unusual because of the clinical presentation and diagnostic challenge. The rare concurrent diagnoses included (1) forearm/hand compartment syndrome, (2) calcium pyrophosphate crystals (pseudogout) in the wrist, (3) monosodium urate crystals, gouty tophi, and *S. haemolyticus* in the elbow, (4) *C. albicans* infection and gouty tophi in the olecranon bursa, and (5) elbow/forearm cellulitis. We initially incorrectly concluded that this was a first-time episode of ACS. Because of this, we focused on the possibility that the crystalline-induced arthritides and concurrent infections had somehow precipitated an atypical episode of ACS.

It is well known that gout attacks typically cause joint pain and swelling. There are only a few cases reported where gout or pseudogout crystal deposition cause high swelling of limb compartments [8]. In these rare cases, the limb compartment swelling usually subsides with rest and anti-inflammatory and/or antiuricemic medications [9–11]. Hence, these patients do not need surgical fasciotomies that are done for compartment syndrome. Our literature review (PubMed/Google Scholar) also revealed no evidence that gout or pseudogout is causally related to ACS or CECS. However, prior to our recognition that our patient had CECS, case reports of carpal tunnel syndrome caused by gouty tophi [12–15] led us to consider the possibility that he had accumulated nonsynovial tophi (e.g., within the fascia) that precipitated ACS by reducing compliance of the fascia. But this hypothesis proved untenable in view of studies showing that tophi do not accumulate significantly in the fascia [5] and the fascia is not rendered thicker or stiffer by gout [16]. Nevertheless, possible additive effects of reduced fascial compliance and impaired venous return have been hypothesized as being causative factors for CECS for some patients [17]. Although gout can be associated with peripheral vascular disease [18], there was also no evidence that our patient had vascular disease.

We also considered the possibility that he had unrecognized metabolic deficiencies. For example, we considered acute rhabdomyolysis as a possible cause of his current episode of compartment syndrome [19, 20], which also led us to consider McArdle disease (glycogen storage disease type V) [21]. However, these conditions were excluded by CK levels that were only mildly elevated at each of the two hospital admissions and several ED visits made by our patient over a four month period. Fabry's disease was also considered as a possible underlying diagnosis because these patients exhibit, similar to our patient, increased levels of uric acid with associated pain and swelling in the distal extremities. Although some of these patients are diagnosed in the third or fourth decade of life, also resembling the age of our patient, his normal levels of *α*-galactosidase A eliminated this possibility [22–24].

Our patient also did not have unrecognized type I diabetes, which has been reported to be causally linked to spontaneous ACS (i.e., no trauma or other cause identified) [25, 26]. We also considered the possibility of a singular episode of ACS caused by Saturday night palsy resulting from prolonged limb ischemia after passing out during surreptitious binge use of alcohol and/or illicit drugs [27]. This possibility was ruled out by our patient's negative blood toxicology screen. Some additional more obscure causes of compartment syndrome include systemic disorders such as nephrotic syndrome, hypothyroidism, viral or drug-induced myositis, systemic capillary leak syndrome, and other rare metabolic disorders [21, 28–30]. These and additional rare causes listed elsewhere [26, 30] were also ruled-out as possible causes.

The concurrent infections seen in our patient suggest an immunocompromised state. Gout is not known to cause an immunocompromised state [31], but it can be misdiagnosed as cellulitis [32]. Our patient's cellulitis was relatively mild when compared to the more severe infections that can precipitate compartment syndrome [33–36]. Type 2 diabetes appears to be the most significant factor contributing to his immunocompromised state [37].

Our realization that our patient had prior episodes of exertional compartment syndrome was serendipitous, occurring after finding reports describing CECS after minimal exertion [1, 38, 39]. Pritchard et al. [38] found that the cause of CECS in 42 patients in the upper extremity (forearm) was rapid or strenuous repetitive tasks, such as keyboard and light assembly work or packing and heavy industrial assembly work (e.g., factory assembly workers). Brown et al. [1] described 12 patients with CECS of the forearm, all of which were treated with fasciotomies. One of their patients who had only partial resolution of symptoms were, like our patient, one with relatively low strenuous activities (46-year-old crane operator and golf player).

With this new information, it became clear that our patient's previous episodes of pain and swelling, which were diagnosed as “gout attacks” by various clinicians in different EDs, were actually multiple episodes of exertional compartment syndrome that occurred during his four-year employment as a construction worker. The episode of compartment syndrome described in this report was his worst even though it was precipitated by less strenuous activities.

Because CECS is rare, the diagnosis is often unclear or delayed [1, 17, 40]. In addition to pain, symptoms can include a feeling of tightness, hardness, or a “pumped up” sensation in the forearm, cramping, swelling, paraesthesiae of the fingers, weakness, and a feeling of loss of control of the hand [1]. The symptoms are often bilateral and are brought on by exertion and relieved by rest, recurring when the precipitating activity is resumed [1]. When a patient with an episode of CECS presents with these and other wide ranging symptoms, they can be—as in our patient—a red herring that distracts healthcare providers from determining the diagnosis in a timely fashion [6, 41]. Adding to the conclusion, the patients are also often asymptomatic at rest, showing minimal findings upon examination. Clinicians need to consider CECS as a cause of activity-induced pain and swelling, and thus diagnostic compartment pressure measurements can be made before, during, and after exertion tests that reproduce the symptoms [6].

## 4. Conclusion

This report describes the rare association of an acute gout attack in a patient that presented to us with what was initially considered to be an isolated episode of ACS. Subsequent workup and more careful consideration of the patient's prior descriptions of some of his “gout attacks” ultimately revealed that he likely had been experiencing CECS. The worst episode of his recurrent CECS of the forearms and hands was the case we describe here. When patients present with ACS without typical causes, occupational/athletic history should be considered to determine if CECS is the cause of symptoms. Clinicians could improve their diagnostic acumen in similar cases by being more specific about the patient's prior episodes of joint pain and swelling versus limb pain and swelling.

## Figures and Tables

**Table 1:** Results of synovial fluid analysis.

	Normal range	Right wrist joint	Right elbow joint
WBC	0–200 mm^3^	No cell count^∗^	91,560 [H]
Crystals	None	CP	MU
RBC	None	No cell count^∗^	21,000
Neutrophils	0–25%	79 [H]	85 [H]
Lymphocytes	0–78%	10	2
Monocytes	0–71%	8	13
Eosinophils	0–2%	3 [H]	None
Appearance	Clear	Bloody	Turbid
Color	Colorless	Red	Yellow

^∗^Sample was clotted. CP = calcium pyrophosphate; MU = monosodium urate; RBC = red blood cell count; WBC = white blood cell count; [H] = high level.

**Table 2:** Thyroid panel.

	Normal range	Result
T3 uptake	23–40%	37
T4	4.6–12 *μ*g/dl	11.8
Free thyroxine index	1.4–4.5 ng/dl	4.366
TSH	0.34–4.82 U/ml	3.81

T3 = serum triiodothyronine; T4 = serum tetraiodothyronine; TSH = thyroid-stimulating hormone.

**Table 3:** General metabolic panels.

	Normal range	Result
Na	136–145 mmol/L	138
K	3.5–5.1 mmol/L	3.9
Cl	98–107 mmol/L	102
CO_2_	23–32 mmol/L	26
Anion gap	4–12 mmol/L	19 [H]
Glucose	70–110 mg/dL	84
BUN	7–18 mg/dL	14
Creatinine	0.6–1.3 mg/dL	0.8
eGFR	>60	>60
PROT	6.4–8.2 g/dL	7.5
Albumin	3.4–5.0 g/dL	2.9 [L]
Calcium	8.5–10.1 mg/dL	8.6
BILI-TOT	0.1–1.2 mg/dL	0.3
AST	5–41 U/L	26
ALT	10–56 U/L	24
ALKPHOS	50–136 U/L	88

ALKPHOS = alkaline phosphatase; ALT = alanine aminotransferase; AST = aspartate aminotransferase; BILI-TOT = bilirubin total; BUN = blood urea nitrogen; Cl = chlorine; CO_2_ = carbon dioxide; eGFR = estimated glomerular filtration rate; K = potassium; Na = sodium; PROT = protein; [H] = high level; [L] = low level.
